# Benefit profile of recombinant human soluble thrombomodulin in sepsis-induced disseminated intravascular coagulation: a multicenter propensity score analysis

**DOI:** 10.1186/s13054-015-0810-3

**Published:** 2015-03-03

**Authors:** Jumpei Yoshimura, Kazuma Yamakawa, Hiroshi Ogura, Yutaka Umemura, Hiroki Takahashi, Miki Morikawa, Yoshiaki Inoue, Satoshi Fujimi, Hiroshi Tanaka, Toshimitsu Hamasaki, Takeshi Shimazu

**Affiliations:** Department of Emergency and Critical Care, Osaka General Medical Center, 3-1-56 Bandai-Higashi, Sumiyoshi-ku, Osaka 558-8558 Japan; Department of Traumatology and Acute Critical Medicine, Osaka University Graduate School of Medicine, 2-15 Yamadaoka, Suita, Osaka 565-0871 Japan; Department of Emergency and Critical Care Medicine, Juntendo University, Urayasu Hospital, 2-1-1 Tomioka, Urayasu, Chiba 279-0021 Japan; Office of Biostatistics and Data Management, National Cerebral and Cardiovascular Center, 5-7-1 Fujishirodai, Suita, Osaka 565-8565 Japan

## Abstract

**Introduction:**

The safety and efficacy of recombinant human soluble thrombomodulin (rhTM) have been demonstrated, with promising evidence suggestive of efficacy for patients with severe sepsis involving coagulopathy in a phase IIb randomized controlled trial. However, the benefit profiles of rhTM have not been elucidated. The purpose of this study was to explore whether patients with greater disease severity, determined according to the Acute Physiology and Chronic Health Evaluation (APACHE) II and Sequential Organ Failure Assessment (SOFA) scores, would experience treatment benefit from rhTM administration.

**Methods:**

This was a *post hoc*, subgroup analysis of a multicenter retrospective cohort study conducted in three Japanese tertiary referral hospitals. Patients with sepsis-induced disseminated intravascular coagulation (DIC) who required ventilator management were included. We stratified patients into several strata according to disease severity, determined by APACHE II and SOFA scores, using classification and regression trees for survival data. Intervention effects, expressed as hazard ratios (HR), were analyzed using Cox regression analysis adjusted for a propensity model to detect subgroup heterogeneity of the effects of rhTM on in-hospital mortality.

**Results:**

Participants were 162 patients with sepsis-induced DIC; 68 of these patients received rhTM and 94 did not. After adjusting for imbalances, rhTM administration was significantly associated with reduced mortality in high-risk patients (APACHE II: 24 to 29; HR: 0.281; 95% confidence interval (CI): 0.093 to 0.850; *P* = 0.025). A similar nonsignificant tendency was observed in the very high-risk subset (APACHE II: ≥30; HR: 0.529; 95% CI: 0.202 to 1.387; *P* = 0.195) but was not evident in the moderate-risk subset of patients (APACHE II: <24; HR: 0.814; 95% CI: 0.351 to 1.884; *P* = 0.630). A similar tendency was observed in analysis of SOFA scores (moderate-risk subset (SOFA: <11), *P* = 0.368; high-risk subset (SOFA: ≥11), *P* = 0.042).

**Conclusions:**

Survival benefit was observed with rhTM treatment in sepsis-induced DIC and high risk of death according to baseline APACHE II and SOFA scores.

## Introduction

Crosstalk between the coagulation system and inflammatory reactions during sepsis causes organ damage followed by multiple organ dysfunction syndrome or death [1-3]. Anticoagulant therapies are therefore expected to be beneficial in the treatment of severe sepsis. Numerous large clinical trials using anticoagulant agents, such as recombinant human activated protein C (rhAPC) [4], tissue factor pathway inhibitor [5], and antithrombin (AT) [6], have been conducted over the past 10 years to evaluate mortality benefit for patients with severe sepsis. While these trials failed to reduce 28-day mortality for all patients with severe sepsis, some subgroup analyses have been conducted to identify patient groups who could benefit from anticoagulant therapy. Consequently, anticoagulant therapies might only be effective for patients with severe sepsis involving disseminated intravascular coagulation (DIC) and high risk of death [7].

Thrombomodulin is a thrombin receptor present on the endothelial cell surface and plays an important role in the regulation of intravascular coagulation [8]. Recombinant human soluble thrombomodulin (rhTM) is widely used in the treatment of DIC in Japan. In a previous study, we demonstrated that rhTM may have a significant beneficial effect on mortality in mechanically ventilated adult patients with sepsis-induced DIC [9]. Moreover, the safety and efficacy of rhTM was demonstrated, with promising evidence of efficacy, in a phase IIb randomized controlled trial and is currently under evaluation in a phase III trial [10]. While the subgroup analysis of the phase IIb trial suggested that patients with respiratory or cardiac dysfunction and coagulopathy characterized by prothrombin time–International Normalized Ratios >1.4 at baseline could benefit from rhTM administration, the benefit profiles have not been thoroughly elucidated.

This study aimed to analyze the efficacy of rhTM treatment in sepsis-induced DIC according to disease severity defined by Acute Physiology and Chronic Health Evaluation (APACHE) II and Sequential Organ Failure Assessment (SOFA) scores.

## Materials and methods

### Study population

This investigation was a *post hoc* subgroup analysis of a multicenter retrospective cohort study conducted in three Japanese tertiary referral hospitals between January 2006 and June 2011 [9]. Inclusion criteria were as follows: infection known or suspected on the basis of clinical data at study entry, two or more signs of systemic inflammation with the presence of sepsis-induced organ dysfunction, hematologic dysfunction (platelet count <80,000/mm^3^), and the need for mechanical ventilation to stabilize the patient’s general condition. All patients fulfilled the criteria for the Japanese Association for Acute Medicine (JAAM) DIC scoring system [11]. The exclusion criteria were as follows: fatal or life-threatening bleeding (intracranial, gastrointestinal, or pulmonary); history of cerebrovascular disorder (cerebral bleeding or infarction) within 1 year; age ≤15 years; history of hypersensitivity to protein preparations or unfractionated heparin; pregnancy or breastfeeding; and fulminant hepatitis, decompensated liver cirrhosis, or other serious liver disorder.

Participants were 162 patients with sepsis-induced DIC who were categorized into one of two groups: the rhTM group, comprising 68 patients who received rhTM; or the control group, comprising 94 patients who received no rhTM. In our retrospective study, there was no predefined protocol regarding definite indications for rhTM treatment. For patients with severe sepsis fulfilling the criteria for DIC, rhTM was used at the discretion of the attending physician. In the rhTM group, rhTM was mainly administered intravenously at a dose of 0.06 mg/kg/day, and infusion continued for 6 days. All patients were treated according to the strategy described in the Surviving Sepsis Campaign Guidelines [12]. We did not administer rhAPC to either group, because it had not been approved for the treatment of severe sepsis in Japan.

This study followed the principles of the Declaration of Helsinki. The study was approved by the Institutional Review Board of the Osaka General Medical Center, and the board waived the need for informed consent for retrospective studies such as this.

### Data collection

Baseline characteristics, including demographic information and data concerning preexisting conditions, organ function/failure, infection, and pertinent medications, were recorded. The variables used to assess comparability between the two groups were age, sex, APACHE II score, SOFA score, number of dysfunctional organs, infection site, and positive blood culture rate.

The primary outcome measure was in-hospital mortality. We also recorded complications including the occurrence of intracranial hemorrhage, gastrointestinal hemorrhage, respiratory tract hemorrhage, and minor transfusions. Minor transfusion was defined as the administration of 4 units of red blood cells or more within two consecutive days for up to 14 days.

### Statistical analysis

**Figure 1:**
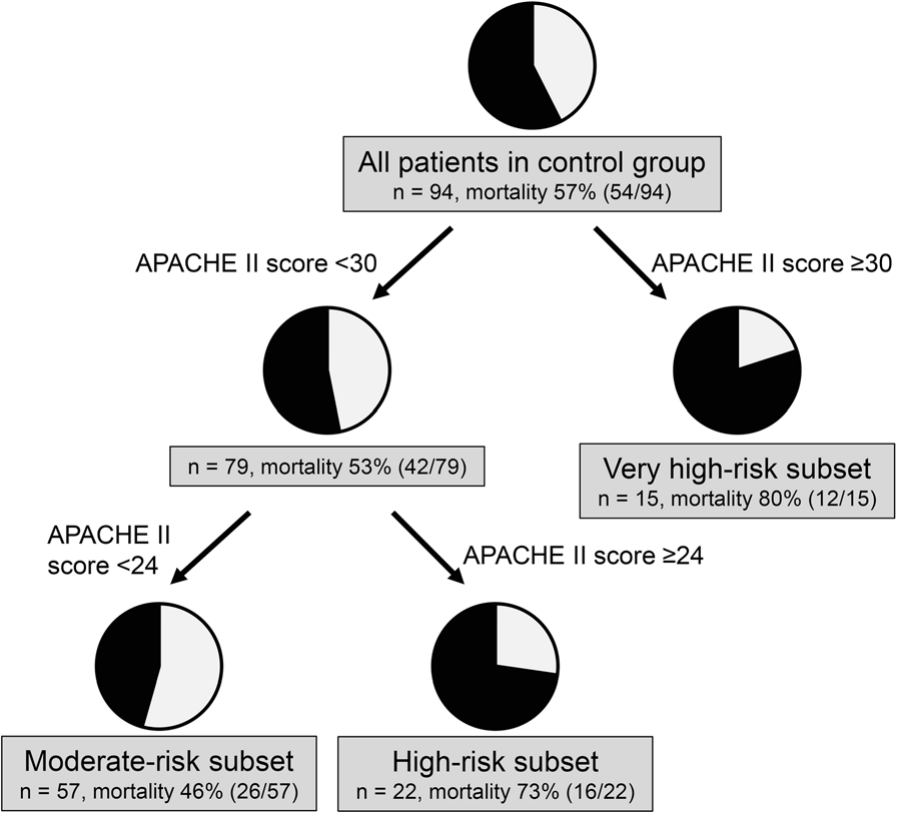
**Control group stratification according to baseline APACHE II scores using the classification and regression tree method.** APACHE, Acute Physiology and Chronic Health Evaluation.

The aim of this study was to identify a subset of patients with high benefit profiles for rhTM treatment. Classification and regression trees for survival data (survival CART) were used to classify patients according to disease severity determined by APACHE II, SOFA, and JAAM DIC scores. Survival CART analysis for APACHE II scores revealed that the first split point at which to partition mortality risk for patients without rhTM treatment was an APACHE II score of 30, and the second split points were APACHE II scores of 24 for all subsets of patients (Figure 1). The intervention effects of rhTM treatment were therefore estimated in three subsets.

Owing to the retrospective nature of the study, there were baseline imbalances between the two groups of patients; therefore, an adjusted mortality analysis was performed using propensity scores [13,14]. The propensity score for receiving rhTM was calculated using multivariate logistic regression and included 15 independent variables, as described in our previous report [9], comprising age, sex, illness severity (APACHE II score, SOFA score, number of dysfunctional organs, and positive blood culture), JAAM DIC score, respiratory dysfunction, time from severe sepsis onset to study entry (defined according to a 48-hour cutoff point), a past medical history of severe conditions (diabetes mellitus and immunosuppressive therapy), source of infection, and therapeutic interventions (heparin administration, AT administration, or emergency operations). The *c* statistic was 0.792. The Hosmer–Lemeshow chi-square value was 9.585 (degrees of freedom = 8), with a nonsignificant *P* value of 0.295, which indicates that the model fit well.

Patients were stratified into quintiles according to propensity score. The overall effectiveness of treatment with respect to mortality was assessed using the Cox regression model with defined strata. Exact method analyses were performed to examine secondary outcomes involving bleeding complications.

Descriptive statistics were calculated as medians (interquartile range) or proportions, as appropriate. Univariate differences between groups were assessed using the Mann–Whitney *U* test, Kruskal–Wallis test, chi-square test, or Fisher’s exact test. *P* <0.05 indicated statistical significance. All statistical analyses were performed using IBM SPSS Statistics version 22.0 for Windows (SPSS Inc., Chicago, IL, USA), SAS Statistical Software version 9.3 for Windows (SAS Institute Inc., Cary, NC, USA), or an in-house validated FORTRAN program.

## Results

### Baseline characteristics

During the study period, 162 consecutive patients fulfilled the inclusion criteria. The rhTM group comprised 68 patients, and the control group comprised 94 patients. Baseline characteristics and therapeutic interventions for the study population are presented in Table 1. Baseline illness severity determined by APACHE II and SOFA scores, numbers of dysfunctional organs, and rates of positive blood culture were significantly higher in the rhTM group relative to the control group. The two groups did not differ significantly with respect to coagulation parameters including DIC scores, past medical history of severe conditions, infection site, and therapeutic intervention. The median duration of rhTM administration was 6.0 (interquartile range 4.0 to 6.0) days, and the median dose of rhTM administration was 0.059 (0.043 to 0.065) mg/kg/day in the rhTM group.

**Table 1:** **Baseline characteristics of all patients with sepsis-induced DIC untreated or treated with rhTM**

	**Overall ** **(** ***n*** **= 162)**	**rhTM group ** **(** ***n*** **= 68)**	**Control group ** **(** ***n*** **= 94)**	***P*** **value** ^**a**^
Patient characteristics				
Age (years)^b^	69 (59 to 76)	69 (61 to 76)	70 (57 to 77)	0.953
Male sex^b^	93 (57%)	36 (53%)	57 (61%)	0.339
Illness severity				
APACHE II score^b^	23 (19 to 29)	25 (21 to 32)	22 (18 to 27)	0.008
SOFA score^b^	11 (9 to 13)	12 (9 to 13)	11 (8 to 12)	0.029
Number of dysfunctional organs^b^	4 (3 to 5)	4 (3 to 5)	4 (3 to 5)	0.383
Positive blood culture^b^	72 (44%)	41 (60%)	31 (33%)	0.001
Coagulation parameters				
Platelet count (/mm^3^)	4.9 (2.7 to 6.5)	4.4 (2.6 to 6.4)	5.3 (2.8 to 6.6)	0.081
PT-INR	1.40 (1.23 to 1.70)	1.40 (1.20 to 1.67)	1.50 (1.30 to 1.78)	0.169
FDP (μg/ml)	22.3 (11.0 to 55.5)	24.6 (13.2 to 60.0)	20.3 (10.2 to 48.9)	0.380
Fibrinogen level (mg/dl)	350 (224 to 495)	357 (225 to 553)	328 (213 to 456)	0.231
JAAM DIC score^b^	6 (5 to 8)	6 (5 to 8)	6 (5 to 8)	0.555
ISTH DIC score	4 (3 to 5)	4 (4 to 5)	4 (3 to 5)	0.457
Organ failure				
Respiratory^b^	114 (70%)	54 (79%)	60 (64%)	0.037
Circulatory	134 (83%)	57 (84%)	77 (82%)	0.835
Kidney	86 (53%)	37 (54%)	49 (52%)	0.873
Metabolic	96 (59%)	40 (59%)	56 (60%)	1.000
Hematologic	162 (100%)	68 (100%)	94 (100%)	1.000
Time from severe sepsis onset to study entry^b^				0.689
Early (≤48 hours)	114 (70%)	49 (72%)	65 (69%)	
Late (>48 hours)	48 (30%)	19 (28%)	29 (31%)	
Co-morbidities				
Diabetes^b^	32 (20%)	17 (25%)	15 (16%)	0.167
Hypertension	38 (24%)	18 (27%)	20 (21%)	0.458
Hemodialysis	9 (6%)	2 (3%)	7 (7%)	0.306
Immunosuppression^b^	19 (12%)	11 (16%)	8 (9%)	0.146
Malignant disease	12 (7%)	5 (7%)	7 (7%)	1.000
Infection site^b^				0.100^c^
Lung	32 (20%)	10 (15%)	22 (23%)	
Abdomen	62 (38%)	23 (34%)	39 (42%)	
Soft tissue	31 (19%)	13 (19%)	18 (19%)	
Urinary tract	21 (13%)	14 (21%)	7 (7%)	
Other/unknown	16 (10%)	8 (12%)	8 (9%)	
Therapeutic interventions				
Vasopressor	131 (81%)	57 (84%)	74 (79%)	0.429
Steroid	49 (30%)	16 (24%)	33 (35%)	0.123
Heparin/heparinoid^b^	18 (11%)	4 (6%)	14 (15%)	0.081
Antithrombin^b^	16 (10%)	9 (13%)	7 (7%)	0.288
Renal replacement therapy	48 (30%)	20 (29%)	28 (30%)	1.000
Emergency operation^b^	63 (39%)	20 (29%)	43 (46%)	0.050

APACHE, Acute Physiology and Chronic Health Evaluation; DIC, disseminated intravascular coagulation; FDP, fibrin/fibrinogen degradation products; ISTH, International Society on Thrombosis and Hemostasis; JAAM, Japanese Association for Acute Medicine; PT-INR, prothrombin time–International Normalized Ratio; rhTM, recombinant human soluble thrombomodulin; SOFA, Sequential Organ Failure Assessment. Data are expressed as group median (interquartile range) or proportion (%). ^a^ *P* value for rhTM-treated patients versus untreated patients. ^b^The 15 variables used in propensity score calculation. ^c^Only one *P* value, for site of infection, is shown because the test was performed as a chi-square test on a 2 × 5 crosstable.

Table 2 presents the baseline characteristics of three subsets stratified according to APACHE II scores using survival CART analysis. Patient characteristics, such as age and sex, were similar between the three APACHE II score subsets. Illness severity, as indicated by APACHE II and SOFA scores, number of dysfunctional organs, and International Society of Thrombosis and Hemostasis (ISTH) DIC scores, showed a significant gradual increase with rising APACHE II scores. Time from onset of severe sepsis to study entry was significantly shorter in the two high-risk subsets relative to the moderate-risk subset.

**Table 2:** **Baseline characteristics of different subsets stratified by baseline APACHE II score**

	**Moderate risk ** **(** ***n*** **= 86)**	**High risk ** **(** ***n*** **= 41)**	**Very high risk ** **(** ***n*** **= 35)**	***P*** **value** ^**a**^
Patient characteristics				
Age (years)	67 (57 to 76)	69 (58 to 76)	72 (66 to 78)	0.171
Male sex	50 (58%)	26 (63%)	17 (49%)	0.419
Illness severity				
APACHE II score	19 (16 to 21)	27 (25 to 28)	33 (32 to 36)	<0.001
SOFA score	9 (7 to 11)	12 (11 to 13)	13 (11 to 15)	<0.001
Number of dysfunctional organs	3 (2 to 4)	4 (3 to 5)	5 (4 to 5)	<0.001
Positive blood culture	31 (36%)	23 (56%)	18 (51%)	0.067
JAAM DIC score	6 (5 to 8)	6 (5 to 8)	8 (6 to 8)	0.092
ISTH DIC score	4 (3 to 5)	4 (4 to 5)	5 (4 to 5)	0.018
Time from severe sepsis onset to study entry				0.035
Early (≤48 hours)	53 (62%)	33 (81%)	28 (80%)	
Late (>48 hours)	33 (38%)	8 (20%)	7 (20%)	
Infection site				0.457^b^
Lung	17 (20%)	6 (15%)	9 (26%)	
Abdomen	35 (41%)	16 (39%)	11 (31%)	
Soft tissue	19 (22%)	9 (22%)	3 (9%)	
Urinary tract	9 (11%)	5 (12%)	7 (20%)	
Other/unknown	6 (7%)	5 (12%)	5 (14%)	

APACHE, Acute Physiology and Chronic Health Evaluation; DIC, disseminated intravascular coagulation; ISTH, International Society on Thrombosis and Hemostasis; JAAM, Japanese Association for Acute Medicine; SOFA, Sequential Organ Failure Assessment. Data are expressed as group median (interquartile range) or proportions (%). ^a^ *P* value between three subsets in the Kruskal–Wallis or chi-square test. ^b^Only one *P* value, for site of infection, is shown because the test was performed as a chi-square test on a 3 × 5 crosstable.

### Effect of treatment on mortality according to baseline disease severity

**Figure 2:**
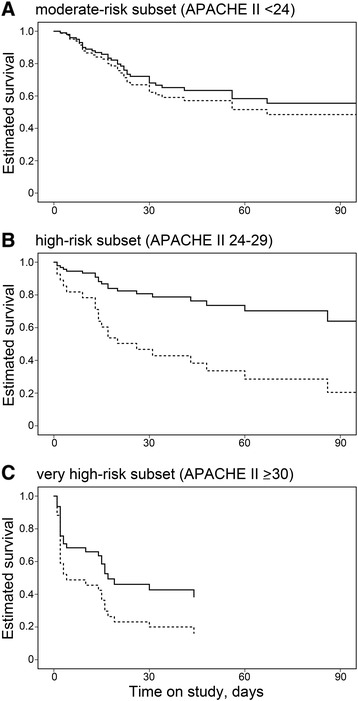
**Adjusted estimated survival curves in subsets stratified according to baseline APACHE II scores. (A)** Moderate-risk subset of patients (APACHE II score <24). **(B)** High-risk subset (APACHE II score = 24 to 29). **(C)** Very high-risk subset (APACHE II score ≥30). Solid line, patients in the rhTM group; dotted line, patients in the control group. Administration of rhTM was only associated with significantly reduced mortality in patients in the high-risk subset (APACHE II score = 24 to 29; *P* = 0.025, Cox regression analysis). APACHE, Acute Physiology and Chronic Health Evaluation; rhTM, recombinant human soluble thrombomodulin.

The survival curves in the prediction model, according to covariates of propensity scores for subsets determined by baseline APACHE II scores, are shown for the rhTM and control groups in Figure 2. Cox regression analysis suggested that rhTM administration was significantly associated with reduced mortality, but only in patients in the high-risk subset (APACHE II score = 24 to 29; adjusted hazard ratio = 0.281; 95% confidence interval = 0.093 to 0.850; *P* = 0.025). In addition, a similar but nonsignificant tendency was observed in the very-high-risk subset of patients (APACHE II score ≥30; hazard ratio = 0.529; 95% confidence interval = 0.202 to 1.387; *P* = 0.195); however, this was not evident in the moderate-risk subset of patients (APACHE II score <24; hazard ratio = 0.814; 95% confidence interval = 0.351 to 1.884; *P* = 0.630).

**Figure 3:**
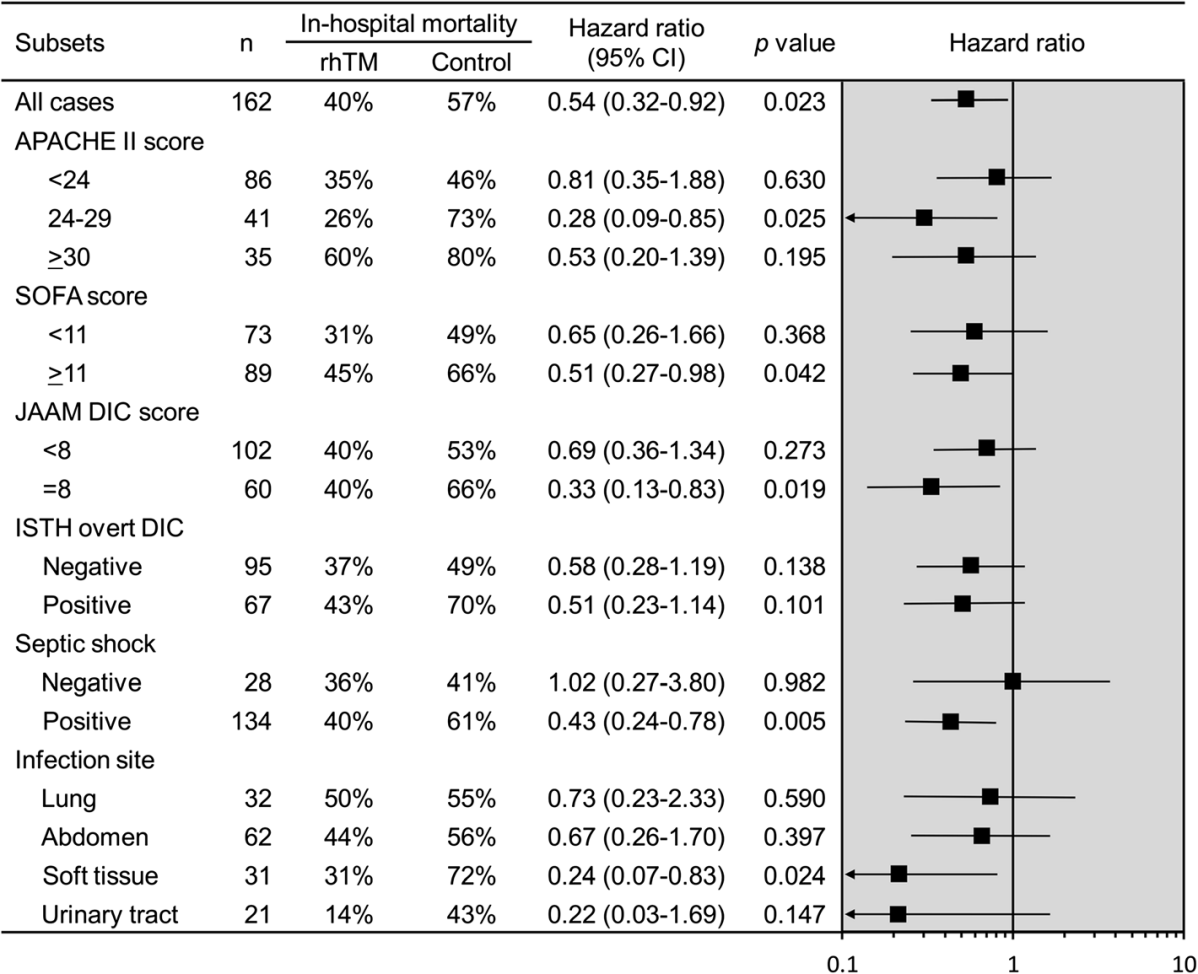
**In-hospital mortality across subsets defined according to measures of baseline disease severity and infection characteristics.** APACHE, Acute Physiology and Chronic Health Evaluation; CI, confidence interval; DIC, disseminated intravascular coagulation; ISTH, International Society of Thrombosis and Hemostasis; JAAM, Japanese Association for Acute Medicine; rhTM, recombinant human soluble thrombomodulin; SOFA, Sequential Organ Failure Assessment.

A similar tendency was also observed in the analysis of subsets based on a number of other clinical measures of baseline disease severity (Figure 3). When the population was separated into subsets according to SOFA scores, the favorable rhTM treatment effect was only observed in the subset with SOFA scores ≥11. With respect to DIC severity, the favorable rhTM treatment effect was only observed in subsets with high DIC severity (JAAM DIC score = 8). In contrast, similar mortality rates were observed in ISTH overt DIC classes with rhTM therapy.

### Adverse events

**Figure 4:**
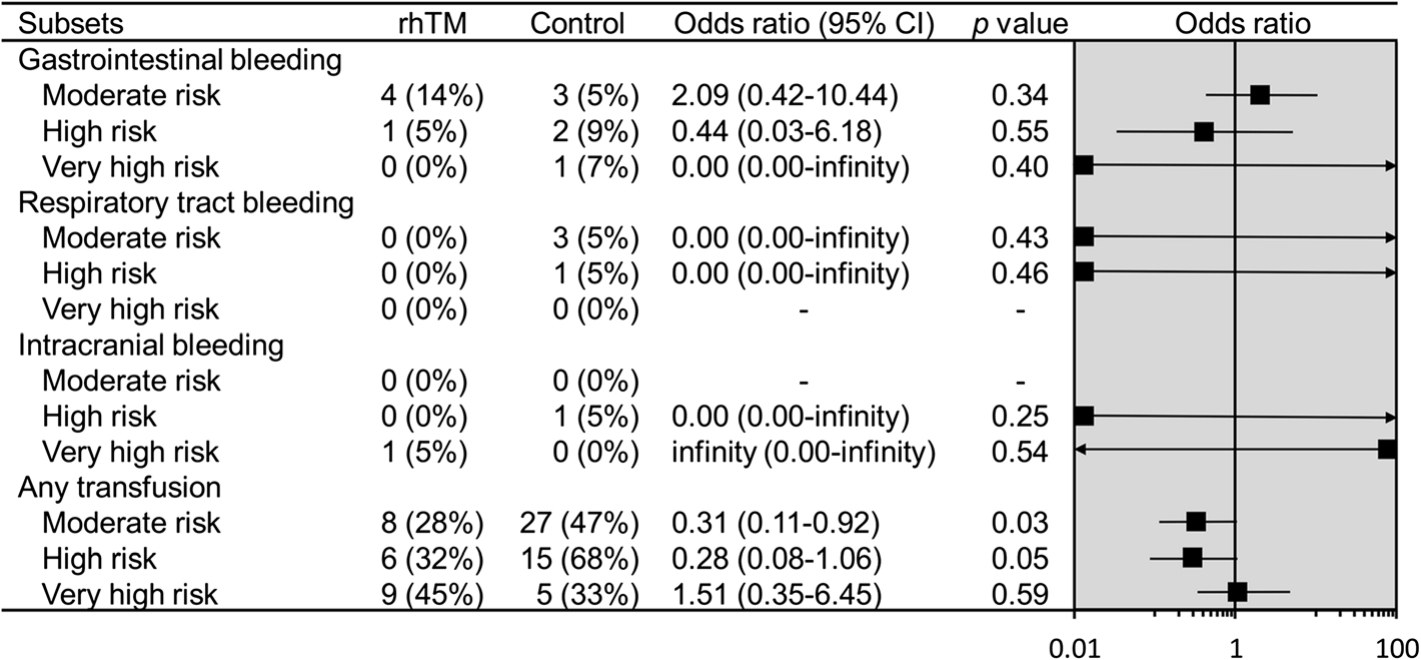
**Bleeding complications in subsets stratified according to baseline APACHE II scores.** APACHE, Acute Physiology and Chronic Health Evaluation; CI, confidence interval; rhTM, recombinant human soluble thrombomodulin.

Bleeding complications in baseline APACHE II score subsets are presented in Figure 4. Rates of minor transfusion and gastrointestinal, respiratory tract, and intracranial bleeding were similar in the treated and untreated groups. However, analysis of bleeding complications was insufficient because of the small sample sizes of the subsets.

## Discussion

The present study represents the first attempt to evaluate the benefit profile of rhTM in sepsis-induced DIC. The results of this study provide evidence that rhTM improved mortality in a high-risk subset (APACHE II score = 24 to 29) of patients with sepsis-induced DIC. The current analysis also indicates that patients in the moderate-risk subset (APACHE II score <24) may not have benefited from rhTM administration. Similar tendencies, with respect to mortality benefit, have been evaluated in analyses of other anticoagulant therapies. The subgroup analysis of the Recombinant Human Activated Protein C Worldwide Evaluation in Severe Sepsis trial suggested increasing absolute and relative risk reduction in rhAPC treatment with greater risk of death, using higher APACHE II scores (≥25) and greater incidence of organ failure [15]. In addition, AT treatment improved mortality in patients with severe sepsis and a predicted mortality of 30 to 60% in the KyberSept trial subgroup analysis [7]. These previous analyses suggest that anticoagulant therapies may only be effective for patients with severe sepsis involving a high risk of death.

Why are anticoagulant therapies for severe sepsis only effective for patients with a high risk of death? Generally, it is difficult to determine the survival benefit of a particular lifesaving therapy in a set of patients with a low risk of mortality. This may be one of the reasons why rhTM administration does not reduce the mortality risk in patients who are not at high risk in the first place. On the other hand, these results are congruent with recent pathophysiological findings concerning the innate immune response. Under certain circumstances, thrombosis is considered to play a major physiological role, which is specifically named immunothrombosis, in immune defense [16]. However, aberrant or uncontrolled activation of immunothrombosis is likely to constitute a key event in the development of thrombotic disorders [2]. In patients in the moderate-risk subset, rhTM could have inhibited host-defensive thrombosis, which helps to capture and ensnare pathogens circulating in the blood, and therefore failed to improve mortality. In contrast, immunothrombosis could have been aberrantly activated and proved detrimental to the host in patients in the high-risk and very-high-risk subsets, which may have improved mortality.

Although several DIC criteria have been established [11,17], whether one criterion performs better than others in detecting patients who require anticoagulant therapies has not been elucidated. In a double-blind randomized controlled phase IIb study [10], mortality benefit was not observed in patients with ISTH overt DIC. In addition, in the current analysis there was no statistically significant correlation between ISTH DIC scores and mortality; however, mortality benefit was observed with rhTM in DIC patients with JAAM DIC scores of 8.

Bleeding was the most significant adverse event associated with rhTM administration, as well as other anticoagulant agents. Indeed, there were more bleeding events with activated protein C and AT relative to a placebo in previous studies [6,18]. Rates of bleeding events were reassessed with respect to illness severity based on APACHE II scores, and there was no increase in bleeding-related adverse events in illness of any severity. However, the subsets were too small to allow observation of adverse effects.

We acknowledge several limitations to our study, which were mentioned in the original paper [9]. The study was not a randomized controlled trial. The relatively long time span of the study could have been associated with the introduction of therapeutic measures that influenced the outcome independently of rhTM. With the study being retrospective, the treatment intervention being examined was not standardized (for example, dose and durations of rhTM administration), and the baseline characteristics were different between the two groups. In the present study, we developed a propensity score approach to cope with the nonrandomization. The combination of these limitations might cause multiple unmeasured variables to account for the outcome differences observed in this study. Also, as this study involved subgroup analysis, we cannot deny the potential of accidental false positive results. Some interventions against sepsis such as rhAPC failed to show a mortality benefit in subsequent large randomized controlled trials [4], although the patients were highly selected according to the positive data of subgroup analysis in previous trials. The study was also prone to false negative results due to inadequate power with which to uncover treatment effect differences, even in the presence of true treatment-effect modification. Further multicenter prospective randomized trials are required to evaluate efficacy and safety distinctly.

## Conclusions

We observed a survival benefit with rhTM treatment in sepsis-induced DIC patients with baseline APACHE II scores ≥24 or SOFA scores ≥11. These findings suggested an increase in treatment benefit with greater risk of death and indicated that application of rhTM treatment may only be limited to sepsis patients with a high risk of death in clinical settings.

## Key messages

- We identified a subset of patients with high benefit profiles for rhTM treatment by stratifying the study sample according to APACHE II and SOFA scores, using survival CART analysis.
- Survival benefit was only observed with rhTM treatment for sepsis-induced DIC in a high-risk subset of patients with APACHE II scores ≥24 or SOFA scores ≥11.
- The results suggested that the survival benefit of rhTM administration may only be experienced by patients with sepsis-induced DIC involving a high risk of death.

## Abbreviations

APACHE: Acute Physiology and Chronic Health Evaluation
AT: antithrombin
CART: classification and regression trees
DIC: disseminated intravascular coagulation
ISTH: International Society of Thrombosis and Hemostasis
JAAM: Japanese Association for Acute Medicine
rhAPC: recombinant human activated protein C
rhTM: recombinant human soluble thrombomodulin
SOFA: Sequential Organ Failure Assessment
